# Left Clavicular Pain Following Splenic Rupture After Colonoscopy: A Variant of Kehr's Sign?

**DOI:** 10.7759/cureus.109950

**Published:** 2026-05-30

**Authors:** Ajinkya A Kulkarni, Adedapo M Adesokan, Amit Jaiswal, Elamin Elamin, Mohamed Shaban Abouletta

**Affiliations:** 1 Emergency Department, Sherwood Forest NHS Foundation Trust, Mansfield, GBR

**Keywords:** colonoscopy complications, kehr's sign, left shoulder pain, post-colonoscopy complications, referred pain, splenic flexure, spontaneous splenic rupture

## Abstract

Colonoscopy is a widely performed diagnostic and therapeutic procedure in modern gastrointestinal practice and is generally considered safe. However, splenic injury remains a rare but potentially life-threatening complication. We present the case of a 67-year-old woman who developed severe epigastric and left-sided pain six days following an uncomplicated colonoscopy. Notably, the patient reported pain localised to the left clavicular region rather than the classical left shoulder-tip pain associated with Kehr's sign. She also experienced a syncopal episode and presented with hypotension and tachycardia. Computed tomography imaging confirmed splenic rupture with haemoperitoneum. The patient underwent emergency exploratory laparotomy and total splenectomy, with evacuation of approximately 1.1 litres of blood and 700 mL of clots. She required a blood transfusion and postoperative intensive care monitoring but made an uneventful recovery. This case highlights the importance of recognising atypical or imprecisely localised referred pain in splenic injury. While clavicular pain may represent a variation of Kehr's sign due to diaphragmatic irritation, clinicians should interpret such distinctions cautiously. A high index of suspicion is essential for early diagnosis and management.

## Introduction

Colonoscopy remains the cornerstone of colorectal disease diagnosis and management. Approximately 900,000 procedures are performed annually in the United Kingdom [1]. Although the procedure is considered safe, splenic injury remains a recognised but rare complication, with reported incidences between 0.0005% and 0.017% [2]. The first reported case of splenic rupture following colonoscopy was described by Wherry and Zehrer in 1974 [3]. Since then, various case reports and reviews have reiterated its clinical importance [4,5]. The proposed mechanisms of injury include traction on the splenocolic ligament, direct trauma from the colonoscope, and the presence of intra-abdominal adhesions [6]. Clinically, patients often present with abdominal pain, shoulder-tip pain (Kehr's sign), hypotension, or syncope [7-10]. However, atypical referred pain patterns such as left clavicular pain may occur due to diaphragmatic irritation transmitted via the phrenic nerve [8]. Delayed diagnosis is common and may increase the associated morbidity and mortality [11]. Given the increasing number of colonoscopies performed and the frequency of prior abdominal surgeries among older patients, clinicians must remain vigilant for splenic injury, even in atypical presentations.

## Case presentation

A 67-year-old woman with a history of chronic diarrhoea underwent an elective diagnostic colonoscopy. She had a prior medical history of hysterectomy, appendectomy, chronic obstructive pulmonary disease (COPD), hypertension, fibromyalgia, irritable bowel syndrome (IBS), and a thyroid cyst. The colonoscopy was performed uneventfully, with multiple benign polyps excised and sent for histopathology. The patient was discharged without complications or reported pain. Available records did not document the level of sedation used during colonoscopy or the use of anticoagulants.

The patient underwent an elective diagnostic colonoscopy (day 0). On the evening of day 6, she developed epigastric pain, left upper quadrant abdominal pain, and pain localised to the left clavicular region. Overnight, the pain progressively worsened and was accompanied by a syncopal episode. On day 7, she presented to the emergency department reporting severe pain (10/10) and appeared clinically unwell. On arrival, her blood pressure was 90/55 mmHg, her pulse rate was 105 beats per minute, her respiratory rate was 34 breaths per minute, her oxygen saturation was 91% on room air, and her temperature was 36.5°C.

The patient was alert and oriented with a Glasgow Coma Scale (GCS) score of 15. Cardiovascular examination revealed tachycardia with a thready pulse and reduced peripheral perfusion, with cool extremities. Respiratory examination demonstrated tachypnoea without focal added sounds. Abdominal examination revealed tenderness in the epigastric and left upper quadrant regions without peritonism.

Her laboratory findings are listed in Table 1.

**Table 1:** Laboratory findings

Parameter	Result	Reference range	Unit
Haemoglobin	109	120-160	g/L
White blood cells	12.2	4.0-11.0	×10^9^/L
Creatinine	95	45-84	µmol/L
Urea	7.9	2.5-7.8	mmol/L
Lactate	1.4	0.5-2.2	mmol/L
Arterial pH	7.42	7.35-7.45	-

Given her haemodynamic instability and recent endoscopic history, intra-abdominal injury was suspected. Computed tomography (CT) of the abdomen and pelvis confirmed a splenic rupture with haemoperitoneum, prompting urgent surgical referral (Figures 1-2).

**Figure 1:**
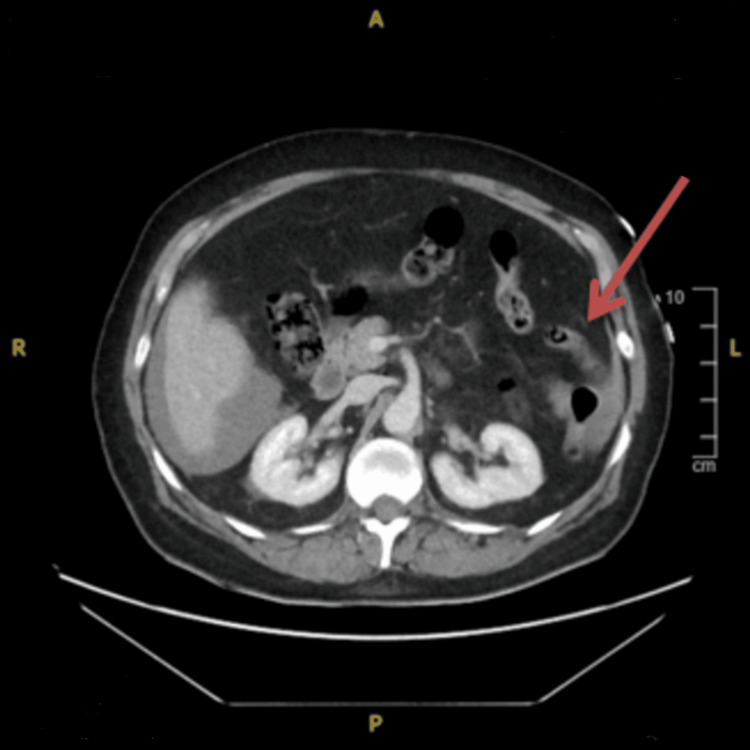
Axial contrast-enhanced computed tomography image of the abdomen demonstrating a significant splenic rupture (red arrow) associated with an acute perisplenic haematoma and free haemoperitoneum

**Figure 2:**
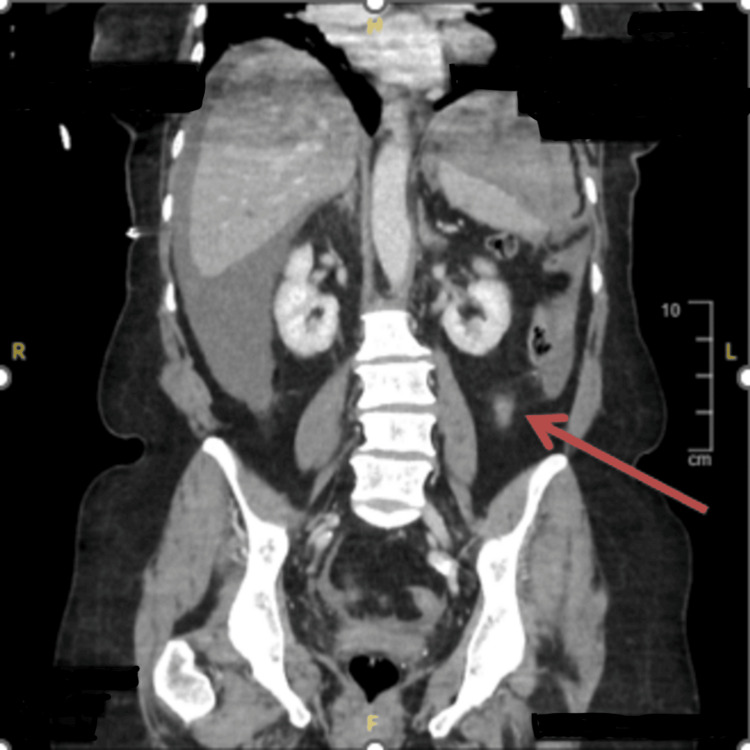
Coronal computed tomography reconstruction of the abdomen and pelvis demonstrating a large volume of free haemoperitoneum pooling within the pelvic cavity

The patient's creatinine was mildly elevated at 95 µmol/L compared to the reference range, likely representing acute pre-renal kidney injury secondary to hypovolaemia from intra-abdominal haemorrhage and haemodynamic compromise. Despite significant haemoperitoneum, lactate and arterial pH remained within normal limits, highlighting that normal lactate does not exclude clinically significant intra-abdominal bleeding.

Initial management included intravenous crystalloid fluid resuscitation, supplemental oxygen therapy, and analgesia. Despite these measures, the patient remained haemodynamically unstable. Although no definite active arterial extravasation was reported on CT imaging, operative management was pursued due to the patient's haemodynamic instability, syncopal episode, and significant haemoperitoneum. In this clinical context, non-operative management or minimally invasive approaches were considered less appropriate than urgent surgical intervention.

The patient underwent emergency exploratory laparotomy. Intraoperatively, a significant haemoperitoneum was identified, with approximately 1.1 litres of free blood and 700 mL of clots evacuated. The spleen was found to be ruptured, with no other intra-abdominal injuries identified. A total splenectomy was performed. She received three units of packed red blood cells intra- and postoperatively. The patient was managed in the intensive care unit for four days and made an uneventful recovery. She was subsequently discharged with appropriate post-splenectomy counselling, including vaccination and infection prophylaxis.

## Discussion

Splenic rupture following colonoscopy remains a rare but significant iatrogenic injury. Recent systematic reviews suggest that its incidence may be underreported, particularly when mild or delayed presentations are managed conservatively [4,6,9]. Abdominal pain is the most common symptom, reported in approximately 46% of patients, while syncope and hypotension occur in around 5% [5,7]. In this case, the patient presented with both hypotension and syncope, despite a normal lactate level. It is important to note that normal lactate does not exclude intra-abdominal haemorrhage [6].

Proposed mechanisms include excessive manipulation or torque of the colonoscope causing traction on the splenocolic ligament, as well as pre-existing adhesions from prior abdominal surgery. Advanced age, female sex, and previous abdominal operations are recognised risk factors [5,8,9]. The delayed presentation in this case may reflect the classical mechanism of delayed splenic rupture. Initial mechanical traction on the splenocolic ligament during colonoscopy may have caused a contained subcapsular splenic haematoma, which gradually expanded over several days before eventual rupture into the peritoneal cavity. This mechanism plausibly explains the patient's delayed symptom onset and subsequent syncopal episode following acute haemodynamic deterioration [12].

Atypical pain referral, as in this case where the patient reported left clavicular pain rather than Kehr's sign, highlights an important diagnostic pitfall. Diaphragmatic irritation may project pain to neighbouring dermatomes through shared cervical nerve roots (C3-C5) [8].

However, it is important to acknowledge that patient-reported localisation of referred pain may be imprecise. The distinction between clavicular pain and classical Kehr's sign may therefore not represent a truly separate clinical phenomenon, but rather a variation in patient perception, clinician interpretation, or documentation. Factors such as communication, questioning technique, and anatomical variability may influence how pain is described. This highlights an important clinical point: any left upper quadrant, shoulder, or adjacent referred pain following colonoscopy should raise suspicion for splenic injury, regardless of whether it conforms to classical descriptions.

A limitation of this report is the absence of detailed intraoperative records and representative intraoperative images, as the case was managed at a different institution with non-digitalised records. Inclusion of these would have further enhanced the educational value of the case.

## Conclusions

This case illustrates an atypical presentation of splenic rupture following colonoscopy, with pain reported in the clavicular region rather than the classical shoulder-tip location. However, given the potential imprecision in patient-reported localisation of referred pain, this distinction should be interpreted cautiously. Clinicians should maintain a high index of suspicion for splenic injury in any patient presenting with abdominal or referred pain following colonoscopy, particularly in the presence of haemodynamic instability. Early imaging with CT remains essential for diagnosis and timely management.
